# Effects of Transcutaneous Electrical Nerve Stimulation on Proinflammatory Cytokines: Systematic Review and Meta-Analysis

**DOI:** 10.1155/2018/1094352

**Published:** 2018-04-02

**Authors:** Tábata Cristina do Carmo Almeida, Francisco Winter dos Santos Figueiredo, Valter Cordeiro Barbosa Filho, Luiz Carlos de Abreu, Fernando Luiz Affonso Fonseca, Fernando Adami

**Affiliations:** ^1^Epidemiology and Data Analysis Laboratory, ABC Medical School (FMABC), Av. Lauro Gomes 2000, Vila Sacadura Cabral, Santo André, 09060-870 São Paulo, SP, Brazil; ^2^Federal Institute of Education, Science and Technology of Ceara, Boa Viagem Campus, Rod. Pres. Juscelino Kubitschek, 63870-000 Boa Viagem, CE, Brazil; ^3^Study Design and Scientific Writing Laboratory, ABC Medical School (FMABC), Av. Lauro Gomes 2000, Vila Sacadura Cabral, Santo André, 09060-870 São Paulo, SP, Brazil; ^4^Clinical Laboratory, ABC Medical School (FMABC), Av. Lauro Gomes 2000, Vila Sacadura Cabral, Santo André, 09060-870 São Paulo, SP, Brazil

## Abstract

The mechanism of pain reduction involves bidirectional processes of pain blocking (nociception) and reductions in the levels of proinflammatory cytokines in the blood. Does transcutaneous electrical nerve stimulation (TENS) reduce blood levels of proinflammatory cytokines? For this systematic review, we searched in six databases to identify randomized controlled trials with the criteria: humans older than 18 years (adults), use of TENS in the experimental group, and having at least one pre- and postintervention blood level of at least one proinflammatory cytokine. The risk of bias and the level of evidence were assessed. Five studies were included involving 240 participants. The heterogeneity of the studies was high (*I* ^2^: 85%); therefore, we used a random-effects meta-analysis. It was observed through the meta-analysis synthesis measures that there were statistically significant differences following the use of TENS to reduce the general group of cytokines. When grouped by chronic disease, by postoperative settings, or by individual studies in the case of IL-6, it was observed that the significant reduction of cytokines related to the use of TENS was maintained. The use of TENS reduced the blood levels of proinflammatory cytokines (we observed a protective factor of TENS in relation to inflammation). The protocol of the systematic review was registered in PROSPERO, CRD42017060379.

## 1. Introduction

Pharmacological therapies for pain and inflammation are recognized and accepted by international medical guidelines as the first line of treatment. However, due to intolerable side effects (e.g., gastritis, nausea, and vomiting) [1] or the ineffectiveness of these interventions in some individuals, analgesic and nonpharmacological treatments with minimal side effects are necessary [2]. In this case, transcutaneous electrical nerve stimulation (TENS) is a physiotherapeutic resource that has been increasingly studied as an alternative therapy.

TENS has been used since 1970 as adjunctive therapy for acute and chronic pain management in various medical and surgical conditions [3]. Currently, evidence on the efficacy of TENS in clinical practice has not yet provided a definitive conclusion as to the clinical effects TENS is capable of generating, whether analgesic or nonanalgesic [1, 4].

Regarding analgesia, studies [1–5] show a reduction in pain intensity when compared to control groups in a variety of diseases. However, the evidence on the best parameters for application and their dose-response relationships in disease-related outcomes (e.g., in low back pain, osteoarthritis, and cancer) are not yet definitive, mainly due to the different clinical protocols performed.

Although TENS has been shown to be effective in controlling pain in several pathologies or after several surgical procedures [3], in some situations, such as thoracotomy or some types of cancer, the control of pain remains controversial [6].

In an attempt to understand the mechanisms of pain and the possible effectiveness of TENS in enabling increasingly effective treatments, recent studies have noted that in addition to analgesia, TENS may have an effect on the circulatory [7, 8], healing [9, 10], and inflammatory systems [6, 11].

In addition to the discovery of these systemic effects (which may be useful for treatments when accessibility to treatment sites is limited or for allowing other nonanalgesic approaches to TENS use), research indicates that the relationship between pain and inflammation is a possible mechanism explaining persistent pain [12–14]. In various clinical situations (e.g., osteoarthritis [15], postoperative recovery [14, 16], and breast cancer [17, 18]), the present proinflammatory cytokines are described as some of the main mediators of the pain process [18, 19].

Inflammatory mediators, once released, promote a change in the peripheral transduction mechanisms of the pain stimulus, leading to a change in the pain perception threshold. When a cascade of proinflammatory cytokines is initiated by a given stimulus, it induces the production and secretion of later or distal cytokines that perpetuate the inflammatory response. They cause sensitization of nociceptors, and in interaction with the central nervous system, they increase the perception of pain (hyperalgesia) [19, 20].

After pain is present, there are two mechanisms of action that may be involved in an analgesia mechanism: (i) nociception blockade that reduces the production of proinflammatory cytokines or (ii) a reduction of proinflammatory cytokines that decreases pain intensity [14, 19, 21].

In this case, TENS therapy acts on afferent nerve fibres, blocking nerve transmission of pain—an effect known as gating theory [22]—or stimulating the release of opioids by the central nervous system [22, 23], both of which have been described as mechanisms in pain reduction.

Considering the interaction between pain and inflammation that has been described, what is the evidence regarding the effect of TENS on reducing the levels of proinflammatory cytokines in patients undergoing this therapy? Which current application parameters (frequency, pulse size, and application interface) might cause this? Is there a relationship between pain reduction and a reduction in cytokines?

To fill this gap, we have performed a systematic review that is aimed at analysing the effect of TENS on the reduction in blood levels of proinflammatory cytokines and the relationship of this reduction with a decrease in pain in adults and, secondarily, at assessing how the current application parameters might influence this outcome. The results of this review may contribute to changes in clinical practice (evidence-based physiotherapy) by indicating if and how TENS reduces the levels of proinflammatory cytokines and pain indicators in individuals exposed to this therapeutic resource.

## 2. Methods

### 2.1. Protocol and Registry

A systematic review of clinical trials was developed following the guidelines of the *Preferred Reporting Items for Systematic Reviews and Meta-Analyses Statement* (PRISMA) [24], as presented in Supplementary Material 1. The protocol of the systematic review was registered in the *International Prospective Register of Systematic Reviews* (PROSPERO) on March 28, 2017 (registry number: CRD42017060379). This complete review followed all procedures described in the review protocol [25].

### 2.2. Eligibility Criteria

The details of the search and the eligibility criteria were presented in a previous publication [25]. Articles published in peer-reviewed journals, which are not limited to a specific language or year of publication and which are in accordance to the following criteria (based on the PICOS strategy [26, 27]), were considered eligible as described in Table 1.

### 2.3. Information Sources

In June 2017, after publication of the protocol of this systematic review, articles were searched in six electronic databases (Medline, Scopus, Web of Science, Physiotherapy Evidence Database (PEDro), Cochrane Clinical Trials, and EMBASE).

As a strategy to increase the scope of the search, we consulted the list of references of the articles included in the review, searched clinical trial repositories (Clinical Trials and Brazilian Registry of Clinical Trials), and contacted eligible work authors with results not yet published to obtain data necessary for the analyses that were not available in the original article.

### 2.4. Search Strategy

The search strategy of the studies was established based on 4 groups of descriptors: population, intervention, type of study, and outcome. The Boolean operator “AND” was used for the combination of groups of descriptors, while the Boolean operator “OR” was used in the combination between the synonyms of the descriptors of the same group.

To improve the search sensitivity, the search strategy for each of the following databases is described and is presented in detail in the supplementary material: Medline (Supplementary Material 2), Scopus (Supplementary Material 3), Web of Science (Supplementary Material 4), Physiotherapy Evidence Database (PEDro; Supplementary Material 5), Cochrane Clinical Trials (Supplementary Material 6), and EMBASE (Supplementary Material 7).

The searches were carried out without the limit of language or year of publication. In the databases that had search filters, we used as limits the type of article (clinical trial), species (humans), and age group (18+ years). The keywords were selected according to the Medical Subject Headings (MeSH) at the National Library of Medicine. The definitions of the biochemical terms for the search were determined after consulting studies that addressed the subject inflammatory cytokines [13, 21, 28–31].

### 2.5. Study Selection

After performing all the searches in the proposed databases, the selection phase was managed by libraries in EndNote X8. The process was performed independently by two reviewers, and in case of disagreement as to the inclusion and exclusion reasons, a third party was consulted until a final consensus was reached.

After the exclusion of duplicates, the selection process was carried out on two levels considering the eligibility criteria: (1) reading of titles and abstracts, with titles excluded for different reasons (Supplementary Material 8), and (2) reading the complete text and excluding studies that did not meet the eligibility criteria (Supplementary Material 9).

In the search of clinical trial repositories, the studies found were read and selected by the same eligibility criteria. The reasons for exclusion are described in Supplementary Material 10.

### 2.6. Data Extraction

Data extraction and management of the included articles were done in duplicate (two reviewers independently). At the end of the entire extraction process, a third reviewer was consulted to resolve disagreements.

The following variables were extracted from the articles considered eligible for the systematic review:

1. Characteristics of the population: age, sex, primary diagnosis, and medical specialty
2. Characteristics of the intervention (TENS parameters): frequency, intensity, pulse duration, modality, application area, duration of treatment, application interface, and follow-up time
3. Biochemical parameters: type of proinflammatory cytokine, evaluation of its expression in blood (pre- and postintervention level), and method of measurement used
4. Methodological information: sample size, secondary outcome, other results, randomization, blinding, and eligibility criteria

### 2.7. Assessment of Risk of Bias and Quality of Evidence

Two independent reviewers assessed the risk of bias in the studies using the clinical trial assessment detailed in the Cochrane Manual of Systematic Reviews [32] and made available in the Review Manager 5.3 (RevMan) program. Disagreements between reviewers were resolved by a third reviewer. For this evaluation, six items of bias risk assessment were considered: randomization, blindness of participants, professionals and evaluators, incomplete outcomes, reports of selective outcomes, and other possible sources of bias. For each item, one can attribute the concept of low risk, high risk, or risk of uncertain bias [32].

### 2.8. Analysis of Results

The level of evidence was set according to the *Grading of Recommendations Assessment, Development and Evaluation* (GRADE) [33–38]. For each outcome of the study, we assessed the quality of the evidence according to the study design, risk of bias [33], inconsistency [36], indirect evidence [37], imprecision [35], and publication bias [34].

The procedures for the meta-analysis were performed using the Review Manager 5.3 program. The effect size (ES) of the intervention with TENS versus IL-1, IL-6, and TNF-*α* was calculated for each study using the standardized mean difference and its respective 95% confidence interval (95% CI), according to the equations used in the software. It was also decided to carry out a meta-analysis on a general effect, since all cytokines are part of the proinflammatory group and since, within a cascade of cytokines, each exerts an influence on the other in production and secretion [20, 21]. The random-effects method was considered for ES estimation [32]. ES was classified according to the Cohen test scale [39] as very small (<0.20), small (0.20 to 0.49), intermediate (0.50 to 0.79), and large (≥0.80).

The variability of the effects of the intervention was tested for statistical heterogeneity using the chi-square test (*χ* ^2^) with the corresponding *p* value (Cochrane test) and through the *I* ^2^ statistic. The heterogeneity of the studies was considered high when *I* ^2^ ≥ 50% [40].

In addition to the general effect, a subgroup analysis was performed to identify ES according to laboratory or clinical characteristics (postoperative or in patients with chronic diseases, i.e., rheumatoid arthritis). A subgroup analysis according to the TENS parameters was not performed, due to the distinctions between the studies in the form of application of the therapy (i.e., different types of frequency and pulse size). An evaluation of publication bias was not performed with graphical/statistical features (e.g., funnel plot) because of the low number of studies included in the meta-analysis [32].

The results are presented in a forest plot chart, arranged in alphabetical order, considering the main author, and in chronological order by year of publication. The results not used in the meta-analysis are described in a narrative synthesis.

## 3. Results

### 3.1. Study Selection

**Figure 1:**
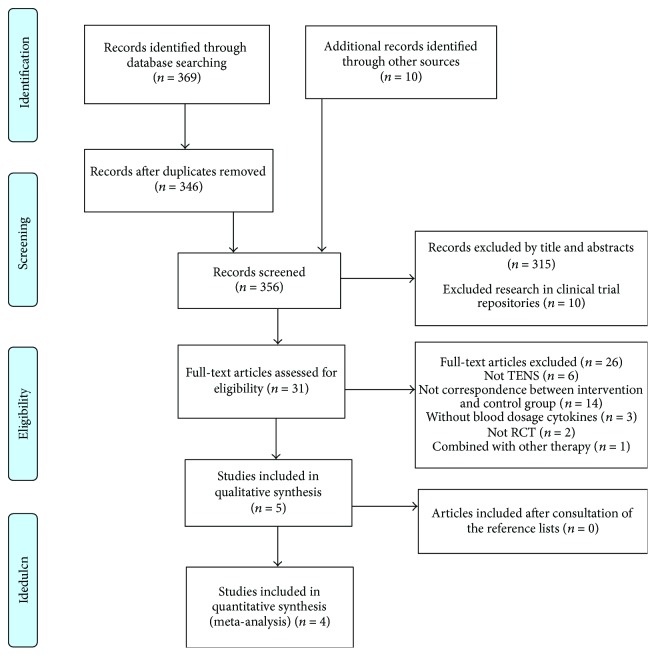
Search process results according to the PRISMA flow diagram

In the initial literature search, we identified 369 publications in databases and 10 publications in clinical trial repositories (Figure 1). After removing duplicates, 356 studies had their titles and abstracts read (exclusion lists of articles after applying the eligibility criteria were presented as Supplementary Materials 8 and 10). After this first step, 31 articles remained to be read in their entirety (exclusion list as Supplementary Material 9). At the end of the research, 5 studies [6, 41–44] were included in the systematic review. Of these, 4 studies [6, 42–44] had data for a cytokine meta-analysis of IL-1 [43], IL-6 [6, 43, 44], and TNF-*α* [6, 42].

### 3.2. Risk of Bias from Individual Studies

**Figure 2:**
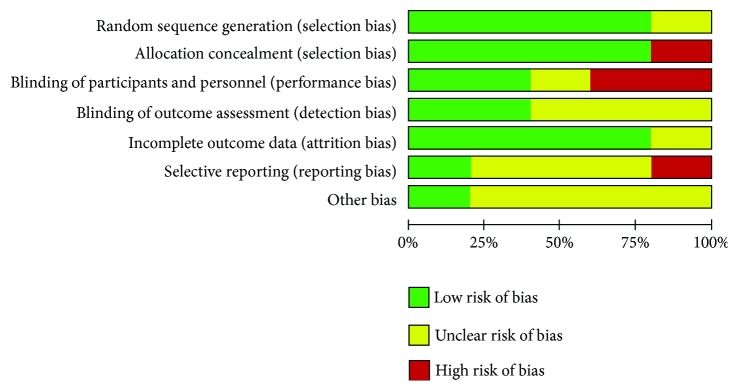
Risk of bias in the studies included in the systematic review.

**Figure 3:**
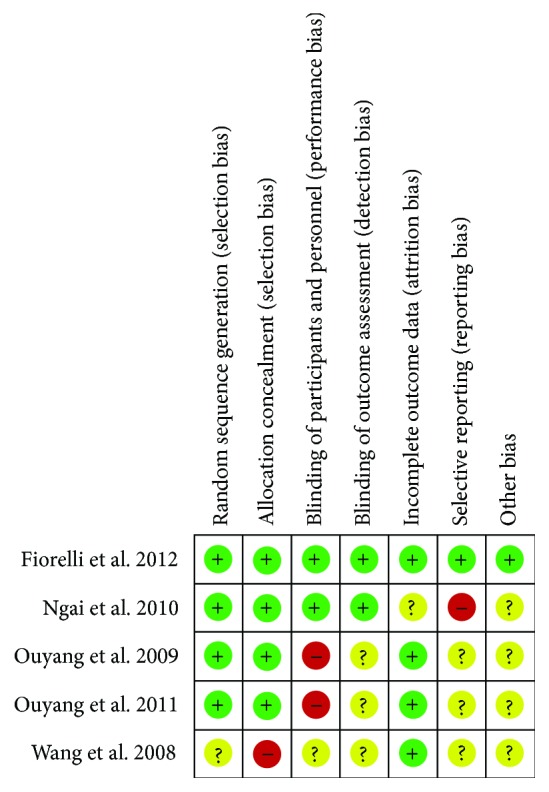
Risk of bias in the individual studies.

The main methodological failures were related to sample size and risk of bias. The main aspects related to risk of bias (Figure 2) were the blindness of participants and evaluators (2 articles) [42, 43], report of a selective outcome (1 study) [45], and concealment of allocation (1 study) [44]. The details of bias risks in each study are available in Figure 3. Despite the specific biases, no studies with a high risk of bias were observed.

### 3.3. Characterization of Studies and Interventions

The general characteristics of the participants, interventions, and outcome studies are presented in Table 2.

In relation to the year of publication, the 5 papers included [6, 42–4] were published from 2008 to 2012. For the language of publication, 2 studies were written in English [6, 41] and 3 studies in Chinese [42–44]. Regarding the countries of origin of the studies, 1 was carried out in Italy [6] and the other 4 studies were carried out in China [41–44].

In total (including intervention and control groups), 240 participants were studied, 108 males and 132 females. The sample size in the studies ranged from 18 [41] to 63 participants [42, 43]. As for the average age, the youngest group was age 49 [42–44] and the older populations were over 73 years old [41].

Regarding TENS parameters, 4 of the 5 studies [41–44] used a needle interface for the application of TENS (electroacupuncture). TENS application parameters (referring to frequency and pulse size) were heterogeneous between the studies.

Regarding the types of proinflammatory cytokines, all were analysed by the ELISA method, and we found as primary outcomes the levels of the following cytokines: IL-1 [43], IL-6 [6, 43, 44], IL-8 [41], and TNF-*α* [6, 41, 42]. Although not used as an outcome in this review, 2 articles [6, 43] presenting levels of IL-10 (anti-inflammatory cytokine) were presented. Pain assessment (secondary outcome) was found only in one article [6].

Regarding the clinical diagnoses found, 3 studies were on [41–43] chronic diseases (rheumatoid arthritis [42, 43] and chronic obstructive pulmonary disease [41]), while 2 studies [6, 44] were in oncological postoperative settings. This information was described in Supplementary Material 11.

Regarding TENS application sites, we found two articles that used the same acupoints [42, 43] (Supplementary Material 11).

### 3.4. Effects of TENS on Proinflammatory Cytokines

Of the included studies, one [41] of five did not report a significant result (*p* > 0.05) for the reduction of IL-8 and TNF-*α*, and this study also did not describe the biochemical results because they did not detect these markers in any subject evaluated. The other four studies [6, 42–44] showed a reduction in cytokine levels (IL-1, IL-6, and TNF-*α*) that was significant in the TENS group, as compared to the control group.

Fiorelli et al. [6] showed significant reductions in IL-6 (*p* = 0.001) and TNF-*α* (*p* = 0.001), and this was the only study of the 5 that evaluated pain by the visual analogue scale. The TENS group had lower pain scores (*p* < 0.001).

Ouyang et al. [43] showed that both IL-1 and IL-6 showed a greater reduction (*p* < 0.05) in the electroacupuncture group when compared to the acupuncture group. The reduction in TNF-*α* in the study by Ouyang et al. [42] was also higher in the TENS group than in the control group (*p* < 0.05). The study of Wang et al. [44] showed a significant reduction in IL-6 in the TENS group in relation to the control (*p* < 0.01).

**Figure 4:**
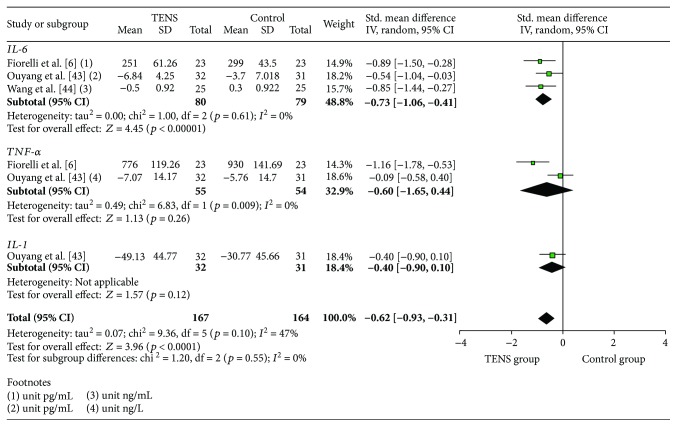
Meta-analysis of the general effect of TENS on proinflammatory cytokines.

Figure 4 presents the results of the meta-analysis in this review regarding the effect of TENS on proinflammatory cytokines. Of the six effect sizes (ES) calculated for the individual studies, four were statistically significant (IL-6 [6, 43, 44] and TNF-*α* [6]). The meta-analysis of the studies indicated that TENS had a significant effect on the reduction of proinflammatory cytokines (standardized mean difference (SMD) = −0.62, 95% CI: −0.93, −0.31). The reduction of IL-6 (3 studies, SMD = −0.73, 95% CI: −1.06, −0.41) was statistically significant in the comparison of participants in the intervention versus the control groups. The TNF-*α* (2 studies, SMD = −0.60, 95% CI: −1.65, 0.44) and IL-1 (1 study, SMD = −0.40, 95% CI: −0.90, 0.10) reductions were not significant. There was a high ES heterogeneity between studies for TNF-*α* (*I* ^2^ = 85%) and a low heterogeneity in the general cytokine group (*I* ^2^ = 47%) and IL-6 (*I* ^2^ = 0%). In the ES classification found in the individual studies, the effect sizes ranged from very small [42] to large [6] (0.09 to 1.16).

**Figure 5:**
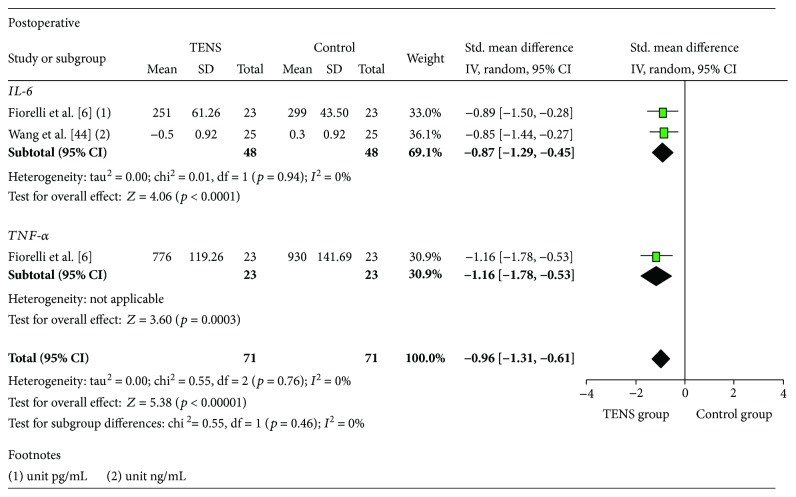
Meta-analysis subgroup postoperative.

In the analysis by subgroups, considering studies of the postoperative period with application of TENS (IL-6 [6, 44] and TNF-*α* [6]) as described in Figure 5, the three calculated ESs were statistically significant. The reduction in this postoperative group was significant, and the size of the achievement was classified as large (SMD = −0.96, 95% CI: −1.31, −0.61). There was heterogeneity between the studies (*I* ^2^ = 0%). It was observed that TENS had a significant effect on IL-6 reduction (2 studies, SMD = −0.87, 95% CI: −1.29, −0.45). The effect on TNF-*α* reduction was also significant (SMD = −1.16, 95% CI: −1.78, −0.53), even though it was evaluated in only one study.

**Figure 6:**
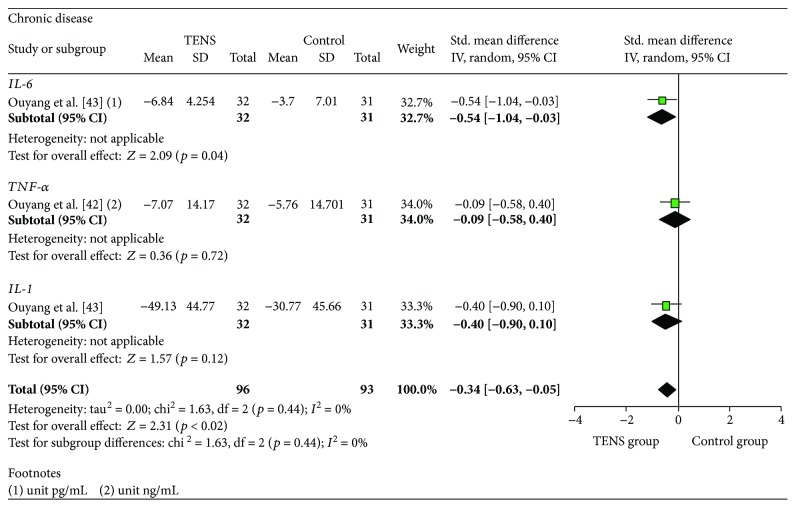
Meta-analysis subgroup chronic disease.

In the subgroup of studies that evaluated TENS in patients with chronic diseases as described in Figure 6, only one study evaluated each outcome: IL-6 [43], TNF-*α* [42], and IL-1 [43]. Only Ouyang et al. [43] found a significant effect of TENS on IL-6 (SMD = −0.54, 95% CI: −1.04, −0.03) in a patient with rheumatoid arthritis. The reduction in this chronic disease group was significant; the size of the achievement was classified as small (SMD = −0.34, 95% CI: −0.63, −0.05) and presented low heterogeneity between studies (*I* ^2^ = 0%).

### 3.5. Assessment of Pain as Outcome

Only one study was found [6] that assessed pain (visual analogue scale (VAS)) as an outcome. The mean pain scores of the TENS group were lower when compared to those of the control group during the entire postoperative period (6 h, 12 h, 24 h, 48 h, 72 h, 96, and 120 h). The difference in VAS scores between the groups was statistically significant (*p* < 0.001).

Without other studies to evaluate the consistency of this effect, it was not possible to determine if there is a relationship between cytokine reduction and pain reduction in the groups after TENS application.

### 3.6. Level of Evidence

The level of evidence of the effect of TENS on proinflammatory cytokines was estimated considering the four outcomes (Table 3). In three of them, a group of proinflammatory cytokines [6, 42–44], IL-6 [6, 43, 44], and TNF-*α* [6, 42] showed a low level of evidence of the effect of TENS on these outcomes. This was due to the low number of studies and inconsistency in results between studies. Regarding the outcome of IL-1 [43], a very low level of evidence was observed. This outcome only was evaluated by one study, and with a small sample size, there was no significant effect of TENS on IL-1.

## 4. Discussion

### 4.1. Risk of Bias on Individual Studies

After evaluation of the included articles, selection bias (allocation concealment), performance bias (participant and professional blinding), and reporting bias (outcome of interest with incomplete data were not used in the meta-analysis) were assessed.

The main problem in assessing TENS in a clinical trial is the blinding method of the subjects involved. Considering that TENS is an electrical current that causes a perceptible sensory stimulus [3], any reporting or questioning that addresses sensation may determine whether the patient is using the rated current. In contrast, biochemical outcomes (proinflammatory cytokines) evaluated by blood levels are unlikely to be influenced by a lack of blinding or identification of allocation in the study, so that this bias does not impact the causality of the study.

On the other hand, studies were concerned with aspects that could compromise the results, such as randomization (less probability of error to clarify a cause-effect relationship between two events) [46] and the description of follow-up losses for a correct analysis fit. Although there are problems with some criteria, the studies did not present a high risk of bias.

### 4.2. Effects of TENS on Proinflammatory Cytokines

The results found in the meta-analysis of the studies showed that even with a high heterogeneity between studies, TENS caused a significant effect on the reduction of proinflammatory cytokines among participants of the intervention group compared to the controls in the general group (intermediate ES). When the results were analysed under specific postoperative conditions (large ES) and chronic diseases (small ES), it was observed that the significant reduction effect was maintained, but there was a reduction in the heterogeneity of the studies. Only IL-6 showed a significant reduction in the three conditions evaluated.

Despite the small number of studies and low levels of evidence, the results were significant probably because they were quantitative and objective measures (cytokine blood levels). The diseases that were evaluated in each study may be the main factor in limiting sample sizes.

In studies evaluating TENS as a strategy for pain control, cytokine levels have been used as an objective measure of the outcome. This is because cytokines play a key role in the acute inflammatory phase and immune response [6].

Levels of IL-6 and TNF-*α* are well known to reflect the degree of surgical trauma because they are markers of the inflammatory response [6, 21]. In this review, the reductions in IL-6 were statistically significant in the postoperative and chronic disease groups. Reductions in TNF-*α* were also observed postoperatively, but as we found a result in only one article, it was not possible to determine the consistency of the effect between studies.

In rheumatoid arthritis, IL-6 has the function of increasing the effect and secretion of IL-1 and TNF-*α* and is also the main marker that is associated with disease activity [42, 43]. The effect of IL-6 reduction, even in a single study, was significant. It should be noted that further research is needed to ensure the consistency of the effect found.

In spite of these findings in a limited number of studies on a reduction effect on proinflammatory cytokines, it is still unknown what mechanism of action is responsible for this effect.

Studies indicate that acupuncture or electroacupuncture facilitates the release of certain neurotransmitters, especially opioids [47]. Release of opioids is also described as the mechanism of action of TENS current by electrodes [16, 22]. Opioids act on the central nervous system and activate the sympathetic or parasympathetic nervous system [22, 47].

An increase in sympathetic activity increases circulating catecholamines in the blood, favouring the reduction of proinflammatory and anti-inflammatory cytokines such as IL-10 [48, 49].

Activation of the parasympathetic nervous system releases acetylcholine (the main neurotransmitter), which by stimulating macrophages can inhibit the production of various proinflammatory cytokines (TNF-*α*, IL-1, and IL-6) [14, 48].

In addition to the mechanisms related to the autonomic nervous system, authors have noted that the pain blocking that occurs at the level of the central nervous system may be responsible for reducing blood levels of proinflammatory cytokines [14, 19, 21, 22].

### 4.3. Strengths and Limitations of the Review

The main limitations of this review are related to sample size (which compromises the accuracy of the data) and a low number of studies (which impacts consistency and does not permit analysis of publication bias). The presence of these limitations directly contributed to the poor quality of the evidence.

Another limitation of importance is the heterogeneity of the clinical protocols, in terms of the evaluated diseases and the TENS application parameters (interface, frequency, and pulse size), making some analyses impossible.

To minimize possible selection biases, the search was extended to assess results not yet published (clinical trial repositories). Even having made these contacts, we could not obtain data with the authors.

As a strength of the review, we emphasize that we have undertaken a comprehensive search including other languages to identify potentially eligible studies. In the case of this review, we included three Chinese articles.

The selection criterion regarding correspondence between the intervention and control groups was established to eliminate possible confounding factors and to ensure that the results were related to TENS.

Another strong point was the comprehensive analysis, considering aspects of methodological quality and the level of evidence of the studies. This allowed us to assess the research implications and clinical practices related to TENS.

### 4.4. TENS Practical and Research Implications

What is known so far is that studies that have observed a pain/cytokine relationship found that patients reporting less severe pain demonstrated a lower production of proinflammatory cytokines [50, 51].

Regardless of the path of this relationship, if the biological effect of TENS is characterized by a significant reduction in cytokine levels, the most important clinical outcome of TENS therapy is pain relief [6].

In the study by Wang et al. [44], for example, which addressed brain surgeries, the use of TENS showed a reduction in cytokines intraoperatively, while the control group showed an increase. Taking into consideration that inhibiting the inflammatory response in the brain (reduction of proinflammatory cytokines) may minimize brain damage, TENS treatments may improve prognosis after surgery [44].

Regarding practical implications, we find that clinically this reduction response of proinflammatory cytokines can be applied in some diseases and bring benefits, mainly in terms of reducing pain and even as a protective factor for inflammation. With more studies proving this effect, we could use TENS at all time points surrounding surgical intervention (pre-, intra-, and postoperative) as a method of decreasing inflammation as well as of helping to control chronic inflammatory diseases such as arthritis and osteoarthritis or even neuropathic pain due to inflammation.

Implications for research on TENS can also be obtained with the present review.

TENS studies have shown several clinical effects. For this reason, it is a resource of physiotherapy that has been applied to various diseases and for different purposes. Clinically, this allows a diversity of approaches, but scientifically, it results in a heterogeneity of methods that hinders some conclusions.

In the case of this review, we were unable to identify which application parameters led to the cytokine reduction effect. As a recommendation for other studies, clinical trials with detailed protocols and groups with defined parameters to be compared (dose-response) are required.

The diversity of scientific methods, the low number of articles, the methodological failures, and the lack of data described did not allow a complete attainment of the objective or a TENS recommendation that has the power of evidence. For further studies, it would be important to calculate the sample size and follow the CONSORT (Consolidated Standards of Reporting Trials) guidelines to reduce methodological failures. Through studies with levels of high evidence, therapies gain power of evidence and their recommendation in clinical practice is strengthened.

In summary, more clinical studies are needed to determine the consistency of the effect, the mechanism of action involved, and the best parameters to optimize dose-responses. Construction of an evidence-based physiotherapy will allow an assessment of the effects of TENS in diseases that would benefit from a reduction in proinflammatory cytokines.

We observed a reduction in proinflammatory cytokines after the use of TENS; however, we did not find strong evidence due to the low number of included articles. Analysing more specifically the largest effect size was observed in the postoperative group, and only IL-6 showed a significant reduction in all the conditions evaluated. We were not able to identify which application parameters (frequency, pulse size, and application interface) led to this effect due to the heterogeneity of the methods of the articles included in this review. Regarding the relationship between cytokine reduction and pain reduction, we did not find enough articles to test the consistency of this relationship.

## Figures and Tables

**Table 1:** Inclusion criteria.

*Study design*
(i) Randomized clinical trials
*Participants*
(i) Humans older than 18 years of age
*Intervention/control*
(i) Transcutaneous electrical nerve stimulation (TENS) (ii) Studies that present the following correspondences between the experimental group and controls to homogenize the effects of the current included the following studies: (1) TENS with application by electrodes × TENS placebo with application of electrodes (2) TENS associated with pharmacological therapy × TENS placebo associated with pharmacological therapy (3) TENS applied by electrode associated with pharmacological therapy × drug therapy (4) TENS applied by needles (electroacupuncture) × acupuncture
*Outcomes*
(i) Primary: blood dosage of proinflammatory cytokines [13, 15, 18, 21] (ii) At least one predose and one TENS intervention of at least one of the major proinflammatory cytokines (iii) Cytokines: interleukin 1*β* (IL-1*β*), interleukin 1*α* (IL-1*α*), interleukin 2 (IL-2), tumor necrosis factor (TNF-*α*), interleukin 6 (IL-6), and interleukin 8 (IL-8) Secondary: pain assessed by the visual analogue scale (VAS)

**Table 2:** Description of the characteristics of the studies.

Author (year)	Participants				Intervention							Outcomes	
	Sex (CG)	Age (CG)	Sex (EG)	Age (EG)	*n* (CG)	*n* (EG)	Application interface	*F* (Hz)	Intensity	Pulse	Modulation	Cytokines	Dosage time
Fiorelli et al. [6]	14 M and 9 F	64 ± 4.1	17 M and 6 F	64 ± 1	23	23	Electrodes	80 Hz	Strong but comfortable	250 *μ*s	Conventional	IL-6, IL-10, and TNF-*α*	Pre, 6 h, 12 h, 24 h, 48 h, 72 h, 96 h, and 120 h
Ngai et al. [41]	8 M and 0 F	71.8 ± 1.9	9 M and 1 F	73.8 ± 2.0	8	10	Needles	2 Hz	Not clear	200 *μ*s	Acupuncture	IL-8 and TNF-*α*	Pre and after 4 weeks
Ouyang et al. [43]	9 M and 22 F	50.5 ± 13.2	9 M and 23 F	49.5 ± 12.9	31	32	Needles	SDZ II type Huatuo	Tolerable by the patient	—	—	IL-1 and IL 6	Pre and 72 h
Ouyang et al. [42]	9 M and 22 F	50.49 ± 13.23	9 M and 23 F	49.52 ± 12.89	31	32	Needles	SDZ II type Huatuo	Tolerable by the patient	—	—	TNF-*α*	Pre and 72 h
Wang et al. [44]	11 M and 14 F	54 ± 10	13 M and 12 F	52 ± 9	25	25	Needles	2/100 Hz	8~12 mA until tolerable by the patient	—	—	IL-6	Pre, 1 h operation, surgery, 24 h after operation, and 48 h

CG: control group; EG: experimental group. M = male; F = female; Hz = hertz; *μ*s = microseconds; mA = milliamperes.

**Table 3:** Methodological quality according to GRADE.

Quality assessment								Effect estimate	Grade score
Number of studies	Study design	Risk of bias	Inconsistency	Indirectness	Imprecision	Other considerations	Publication bias	Absolute (95% CI)	Quality
*Proinflammatory cytokines*									
4	RCT (a)	Serious (−1)	NO (0)	NO (0)	Serious (−1)	None	Unclear, but <10 studies (0)	SMD 0.62 lower (0.93 lower to 0.31 lower)	GRADE score: −2 ⨁⨁◯◯ low quality
*IL-6*									
3	RCT (b)	Serious (−1)	NO (0)	NO (0)	Serious (−1)	None	Unclear, but <10 studies (0)	SMD 0.73 lower (1.06 lower to 0.41 lower)	GRADE score: −2 ⨁⨁◯◯ low quality
*TNF-α*									
2	RCT (c)	Serious (−1)	NO (0)	NO (0)	Serious (−1)	None	Unclear, but <10 studies (0)	SMD 0.6 lower (1.65 lower to 0.44 higher)	GRADE score: −2 ⨁⨁◯◯ low quality
*IL-1*									
1	RCT (d)	Serious (−1)	NO (0)	NO (0)	Very serious (−2)	None	Unclear, but <10 studies (0)	SMD 0.40 lower (0.90 lower to 0.10 higher)	GRADE score: −3 ⨁◯◯◯ very low quality

RCT: randomized clinical trial; CI: confidence interval; SMD: standard mean difference. (a): Fiorelli et al. [6], Ouyang et al. [42], Ouyang et al. [43], and Wang et al. [44]. (b): Fiorelli et al. [6], Ouyang et al. [43], and Wang et al. [44]. (c): Fiorelli et al. [6] and Ouyang et al. [42]. (d): Ouyang et al. [43].

## Abbreviations

ES: Effect size
GRADE: Grading of Recommendations Assessment, Development and Evaluation
IL-1*β*: Interleukin 1*β*
IL-1*α*: Interleukin 1*α*
IL-2: Interleukin 2
IL-6: Interleukin 6
IL-8: Interleukin 8
MeSH: Medical Subject Headings
PRISMA: *The Preferred Reporting Items for Systematic Reviews and Meta-Analyses Statement*
PROSPERO: *International Prospective Register of Systematic Reviews*
RevMan: Review Manager program
SMD: Standardized mean difference
TENS: Transcutaneous electrical nerve stimulation
TNF-*α*: Tumor necrosis factor alpha
VAS: Visual analogue scale.

## Supplementary Materials

Supplementary Materials Supplementary Material 1: checklist PRISMA. Supplementary Material 2: search results Medline (PubMed). Supplementary Material 3: search results Scopus. Supplementary Material 4: search results Web of Science. Supplementary Material 5: search results Physiotherapy Evidence Database (PEDro). Supplementary Material 6: search results Cochrane Clinical Trials. Supplementary Material 7: search results Medline EMBASE. Supplementary Material 8: list of excluded articles (first level: reading title and abstracts). Supplementary Material 9: list of excluded articles (second level: reading the full text). Supplementary Material 10: list of excluded research in clinical trial repositories. Supplementary Material 11: description of other characteristics of the studies.
