# Supplementary material for: Effects of Transcutaneous Electrical Nerve Stimulation on Proinflammatory Cytokines: Systematic Review and Meta-Analysis
Source: Mediators Inflamm. 2018 Apr 2;2018:1094352. doi: 10.1155/2018/1094352 (PMC5901481; doi:10.1155/2018/1094352)
Supplement: Supplementary Materials — Supplementary Material 1: checklist PRISMA. Supplementary Material 2: search results Medline (PubMed). Supplementary Material 3: search results Scopus. Supplementary Material 4: search results Web of Science. Supplementary Material 5: search results Physiotherapy Evidence Database (PEDro). Supplementary Material 6: search results Cochrane Clinical Trials. Supplementary Material 7: search results Medline EMBASE. Supplementary Material 8: list of excluded articles (first level: reading title and abstracts). Supplementary Material 9: list of excluded articles (second level: reading the full text). Supplementary Material 10: list of excluded research in clinical trial repositories. Supplementary Material 11: description of other characteristics of the studies. [file 1094352.f1.pdf]

SUPPLEMENTARY MATERIAL 1: Checklist PRISMA

Section/topic	#	Checklist item	Page/Line
**TITLE**			
Title	1	Identify the report as a systematic review, meta-analysis, or both.	1
**ABSTRACT**			
Structured summary	2	Provide a structured summary including, as applicable: background; objectives; data sources; study eligibility criteria, participants, and interventions; study appraisal and synthesis methods; results; limitations; conclusions and implications of key findings; systematic review registration number.	2
**INTRODUCTION**			
Rationale	3	Describe the rationale for the review in the context of what is already known.	3
Objectives	4	Provide an explicit statement of questions being addressed with reference to participants, interventions, comparisons, outcomes, and study design (PICOS).	4
**METHODS**			
Protocol and registration	5	Indicate if a review protocol exists, if and where it can be accessed (e.g., Web address), and, if available, provide registration information including registration number.	2, 4
Eligibility criteria	6	Specify study characteristics (e.g., PICOS, length of follow-up) and report characteristics (e.g., years considered, language, publication status) used as criteria for eligibility, giving rationale.	5
Information sources	7	Describe all information sources (e.g., databases with dates of coverage, contact with study authors to identify additional studies) in the search and date last searched.	6
Search	8	Present full electronic search strategy for at least one database, including any limits used, such that it could be repeated.	6
Study selection	9	State the process for selecting studies (i.e., screening, eligibility, included in systematic review, and, if applicable, included in the meta-analysis).	7
Data collection process	10	Describe method of data extraction from reports (e.g., piloted forms, independently, in duplicate) and any processes for obtaining and confirming data from investigators.	8
Data items	11	List and define all variables for which data were sought (e.g., PICOS, funding sources) and any assumptions and simplifications made.	8

Risk of bias in individual studies	12	Describe methods used for assessing risk of bias of individual studies (including specification of whether this was done at the study or outcome level), and how this information is to be used in any data synthesis.	8,9
Summary measures	13	State the principal summary measures (e.g., risk ratio, difference in means).	9
Synthesis of results	14	Describe the methods of handling data and combining results of studies, if done, including measures of consistency (e.g., I^2) for each meta-analysis.	9

Section/topic	#	Checklist item	Reported on page #
Risk of bias across studies	15	Specify any assessment of risk of bias that may affect the cumulative evidence (e.g., publication bias, selective reporting within studies).	8
Additional analyses	16	Describe methods of additional analyses (e.g., sensitivity or subgroup analyses, meta-regression), if done, indicating which were pre-specified.	9
**RESULTS**			
Study selection	17	Give numbers of studies screened, assessed for eligibility, and included in the review, with reasons for exclusions at each stage, ideally with a flow diagram.	10
Study characteristics	18	For each study, present characteristics for which data were extracted (e.g., study size, PICOS, follow-up period) and provide the citations.	11,12
Risk of bias within studies	19	Present data on risk of bias of each study and, if available, any outcome level assessment (see item 12).	11
Results of individual studies	20	For all outcomes considered (benefits or harms), present, for each study: (a) simple summary data for each intervention group (b) effect estimates and confidence intervals, ideally with a forest plot.	12
Synthesis of results	21	Present results of each meta-analysis done, including confidence intervals and measures of consistency.	13, 14
Risk of bias across studies	22	Present results of any assessment of risk of bias across studies (see Item 15).	12
Additional analysis	23	Give results of additional analyses, if done (e.g., sensitivity or subgroup analyses, meta-regression [see Item 16]).	14
**DISCUSSION**			
Summary of evidence	24	Summarize the main findings including the strength of evidence for each main outcome; consider their relevance to key groups (e.g., healthcare providers, users, and policy makers).	16
Limitations	25	Discuss limitations at study and outcome level (e.g., risk of bias), and at review-level (e.g., incomplete retrieval of identified research, reporting bias).	18
Conclusions	26	Provide a general interpretation of the results in the context of other evidence, and implications for future research.	20

**FUNDING**			
Funding	27	Describe sources of funding for the systematic review and other support (e.g., supply of data); role of funders for the systematic review.	-----

* Moher D, Liberati A, Tetzlaff J, Altman DG, The PRISMA Group (2009). Preferred Reporting Items for Systematic Reviews and Meta-Analyses: The PRISMA Statement. PLoS Med 6(6): e1000097. doi:10.1371/journal.pmed10

SUPPLEMENTARY MATERIAL 2: Search results Medline (Pubmed)

Number of localized studies: 97
Number of studies after applying limits: 31

	**Descriptors**	**Number of studies reached**
**#1**	adult **OR** adults **OR** human **OR** humans	2017: 17,270,962
**#2**	Transcutaneous Electric Nerve Stimulation **OR** Electrical Stimulation, Transcutaneous **OR** Stimulation, Transcutaneuos Electrical **OR** Transcutaneous Electrical Stimulation **OR** Percutaneuos Electric Nerve Stimulation **OR** Transdermal Electrostimulation **OR** Electrostimulation, Transdermal **OR** TENS **OR** Transcutaneous Electrical Nerve Stimulation **OR** Transcutaneous Nerve Stimulation **OR** Nerve Stimulation, Transcutaneuos **OR** Stimulation, Transcutaneous Nerve **OR** Electric Stimulation, Transcutaneous **OR** Stimulation, Transcutaneous Electric **OR** Transcutaneous Electric Stimulation **OR** Percutaneuos Electrical Nerve Stimulation **OR** Analgesic Cutaneous Electrostimulation **OR** Cutaneous Electrostimulation, Analgesic **OR** Electrostimulation, Analgesic Cutaneous **OR** Electroanalgesia	2017: 19,613
**#3**	“randomized controlled trial” [Publication Type] **OR** “controlled clinical trial” [Publication Type] **OR** “randomized controlled trials” [MeSH Terms] **OR** “random allocation” [MeSH Terms] **OR** “double blind method” [MeSH Terms] OR single blind method [MeSH Terms] **OR** “clinical trial” [Publication Type] OR “clinical trials” [MeSH Terms] **OR** single* [Text Word] **OR** double* [Text Word] **OR** treble* [Text Word] **OR** triple* [Text Word] **OR** placebos [MeSH Terms] **OR** placebo* [Text Word] **OR** random* [Text Word] **OR** “research design” [MeSH Terms] **OR** “comparative study” [MeSH Terms] **OR** “evaluation studies” [MeSH Terms] **OR** follow-up stud* [MeSH Terms] **OR** prospective stud* [MeSH Terms] **OR** control* [Text Word] **OR** prospectiv* [Text Word] **OR** volunteer* [Text Word] **AND NOT** "animals"[MeSH Terms] **AND NOT** ("humans"[MeSH Terms] **AND** "animals"[MeSH Terms])	2017: 4,744,106
**#4**	Chemokines **OR** Cytokines, Chemotactic **OR** Intercrines **OR** Chemotactic Cytokines **OR** cytokines **OR** cytokine.	2017: 709,218
**#5**	**#1** AND **#2** AND **#3** AND **#4**	2017: 97
**#6**	**Limits**: article types (Clinical Trial); Species (humans); age group (19 + years); without limitation of language or year of publication	2017: 31

SUPPLEMENTARY MATERIAL 3: Search results SCOPUS

Number of localized studies: 101

Number of studies after applying limits: 47

	**Descriptors**	Number of studies reached
**#1**	TITLE-ABS-KEY (adult **OR** adults **OR** human **OR** humans)	2017: 19,524,318
**#2**	TITLE-ABS-KEY ("Transcutaneous Electric Nerve Stimulation" OR "Electrical Stimulation, Transcutaneous" OR "Stimulation, Transcutaneuos Electrical" OR "Transcutaneous Electrical Stimulation" OR "Percutaneuos Electric Nerve Stimulation" OR "Transdermal Electrostimulation OR Electrostimulation, Transdermal" OR "TENS" OR "Transcutaneous Electrical Nerve Stimulation" OR "Transcutaneous Nerve Stimulation" OR "Nerve Stimulation, Transcutaneuos" OR "Stimulation, Transcutaneous Nerve" OR "Electric Stimulation, Transcutaneous" OR "Stimulation, Transcutaneous Electric" OR "Transcutaneous Electric Stimulation" OR "Percutaneuos Electrical Nerve Stimulation" OR "Analgesic Cutaneous Electrostimulation" OR "Cutaneous Electrostimulation, Analgesic" OR "Electrostimulation, Analgesic Cutaneous" OR "Electroanalgesia")	2017: 67,931
**#3**	TITLE-ABS-KEY ("randomized controlled trial" **OR** "controlled clinical trial" **OR** "randomized controlled trials" **OR** "random allocation" **OR** "double blind method" **OR** "single blind method" **OR** "clinical trial" **OR** "clinical trials") **OR** TITLE-ABS-KEY (clinical* AND trial*) OR TITLE-ABS-KEY (single* OR double* **OR** treble* **OR** triple* **OR** placebos **OR** placebo* **OR** random* **OR** "research design" **OR** "comparative study" **OR** "evaluation studies" **OR** follow-up stud* **OR** prospective stud* **OR** control* **OR** prospectiv* **OR** volunteer*) **AND NOT** TITLE-ABS-KEY (animal) **AND NOT** TITLE-ABS-KEY (human **AND** animal))	2017: 5,234,452
**#4**	TITLE-ABS-KEY (Chemokines OR "Cytokines, Chemotactic" OR Intercrines OR "Chemotactic Cytokines" OR Cytokines OR Cytokine)	2017: 17,009
**#5**	*SEARCH (COMBINE QUERIES)* **#1** AND **#2** AND **#3** AND **#4**	2017: 101
**#6**	**Limits:** AND (LIMIT-TO (DOCTYPE,"ar")) AND (LIMIT-TO (EXACTKEYWORD,"Controlled Study")) AND (LIMIT-TO (EXACTKEYWORD,"Randomized Controlled Trial"))	2017: 47

SUPPLEMENTARY MATERIAL 4: Search results WEB OF SCIENCE

Number of localized studies: 10
Number of studies after applying limits: 08

	**Descriptors**	Number of studies reached
**#1**	TS= (adult) OR TS=(adults) OR TS=(human) OR TS=(humans)	2017: 4,323,018
**#2**	TS=("Transcutaneous Electric Nerve Stimulation") OR TS=("Electrical Stimulation, Transcutaneous") OR TS=("Stimulation, Transcutaneuos Electrical") OR TS=("Transcutaneous Electrical Stimulation") OR TS=("Percutaneuos Electric Nerve Stimulation") OR TS=("Transdermal Electrostimulation") OR TS=("Electrostimulation, Transdermal") OR TS=(TENS) OR TS=("Transcutaneous Electrical Nerve Stimulation") OR TS=("Transcutaneous Nerve Stimulation") OR TS=("Nerve Stimulation, Transcutaneuos") OR TS=("Stimulation, Transcutaneous Nerve") OR TS=("Electric Stimulation, Transcutaneous") OR TS=("Stimulation, Transcutaneous Electric") OR TS=("Transcutaneous Electric Stimulation") OR TS=("Percutaneuos Electrical Nerve Stimulation") OR TS=("Analgesic Cutaneous Electrostimulation") OR TS=("Cutaneous Electrostimulation, Analgesic") OR TS=("Electrostimulation, Analgesic Cutaneous") OR TS=(Electroanalgesia)	2017: 46,298
**#3**	TS=("randomized controlled trial") OR TS=("controlled clinical trial") OR TS=("randomized controlled trials") OR TS=("random allocation") OR TS=("double blind method") OR TS=("single blind method") OR TS=("clinical trial") OR TS=("clinical trials") OR (TS=(clinical*) AND TS=(trial*)) OR TS=(single) OR TS=(double) OR TS=(treble*) OR TS=(triple*) OR TS=(placebos) OR TS=(placebo*) OR TS=(random*) OR TS=("research design") OR TS=("comparative study") OR TS=("evaluation studies") OR TS=(follow-up stud*) OR TS=(prospective stud*) OR TS=(control*) OR TS=(prospectiv*) OR TS=(volunteer*) NOT TS=(animal) NOT TS=(human AND animal)	2017: 9,675,420
**#4**	TS= (Chemokines) OR TS= ("Cytokines, Chemotactic") OR TS=(Intercrines) OR TS=("Chemotactic Cytokines") OR TS=(Cytokines) OR TS=(Cytokine)	2017: 355,219
**#5**	Search History (combine)<br>**#1** AND **#2** AND **#3** AND **#4**	2017: 10
**#6**	**Limits:** documents types (articles); without limitation of language or year of publication; adults, clinical trials	2017: 08

SUPPLEMENTARY MATERIAL 5: Search results Physiotherapy Evidence Database - *PEDro*

Number of localized studies: 268
Number of studies after applying limits: 268

	**Descriptors**	Number of studies reached
**#1**	Choose the option # Advanced search #	_
**#2**	Abstract & Title: "***transcutaneous electrical nerve stimulation***"	_
**#3**	Therapy: select *"**electrotherapies, heat, cold**"*	_
**#4**	Problem: *Do not select anything*	_
**#5**	Body Part: *Do not select anything*	_
**#6**	Subdiscipline: *Do not select anything*	_
**#7**	Topic: select *"**no appropiate value in this field"***	_
**#8**	Method: select *"**clinical trial"***	_
**#9**	# When searching: select (x) match all search terms (AND)#	268

SUPPLEMENTARY MATERIAL 6: Search results COCHRANE CLINICAL TRIALS

Number of localized studies: 9
Number of studies after applying limits: 9

	**Descriptors**	Number of studies reached
**#1**	(adult):ti,ab,kw **OR** (adults):ti,ab,kw **OR** **(**human):ti,ab,kw **OR** (humans):ti,ab,kw	2017: 824783
**#2**	("Transcutaneous Electric Nerve Stimulation"):ti,ab,kw **OR** ("Electrical Stimulation, Transcutaneous"):ti,ab,kw **OR** ("Stimulation, Transcutaneuos Electrical"):ti,ab,kw **OR** ("Transcutaneous Electrical Stimulation"):ti,ab,kw **OR** ("Percutaneuos Electric Nerve Stimulation"):ti,ab,kw **OR** ("Transdermal Electrostimulation"):ti,ab,kw **OR** ("Electrostimulation, Transdermal"):ti,ab,kw **OR** (TENS):ti,ab,kw **OR** ("Transcutaneous Electrical Nerve Stimulation"):ti,ab,kw **OR** **("**Transcutaneous Nerve Stimulation"):ti,ab,kw **OR** ("Nerve Stimulation, Transcutaneuos"):ti,ab,kw **OR** ("Stimulation, Transcutaneous Nerve"):ti,ab,kw **OR** ("Electric Stimulation, Transcutaneous"):ti,ab,kw **OR** ("Stimulation, Transcutaneous Electric"):ti,ab,kw **OR** ("Transcutaneous Electric Stimulation"):ti,ab,kw **OR** ("Percutaneuos Electrical Nerve Stimulation"):ti,ab,kw **OR** ("Analgesic Cutaneous Electrostimulation"):ti,ab,kw **OR** ("Cutaneous Electrostimulation, Analgesic"):ti,ab,kw **OR** ("Electrostimulation, Analgesic Cutaneous"):ti,ab,kw **OR** (Electroanalgesia):ti,ab,kw	2017: 2065
**#3**	(randomized controlled trial):pt OR (controlled clinical trial):pt OR me (randomized controlled trials) OR me (random allocation) OR me (double blind method) OR me (single blind method) OR (clinical trial):pt OR me (clinical trials) OR (clinical* AND trial*):ti,ab,kw OR (single*):ti,ab,kw OR (double*):ti,ab,kw OR (treble*):ti,ab,kw OR (triple*):ti,ab,kw OR me (placebos) OR (placebo*):ti,ab,kw OR (random*):ti,ab,kw OR ("research design"):ti,ab,kw OR me (comparative study) OR me (evaluation studies) OR me (follow-up stud*) OR me (prospective stud*) OR (control*):ti,ab,kw OR (prospectiv*):ti,ab,kw OR (volunteer*):ti,ab,kw AND NOT (animal):ti,ab,kw AND NOT (human AND animal):ti,ab,kw	2017: 899996
**#4**	(Chemokines):ti,ab,kw **OR** ("Cytokines, Chemotactic"):ti,ab,kw **OR** **(**Intercrines):ti,ab,kw **OR** ("Chemotactic Cytokines"):ti,ab,kw **OR** (cytokines):ti,ab,kw **OR** (cytokine):ti,ab,kw	2017: 9805
**#5**	**#1** AND **#2** AND **#3** AND **#4**	2017: 9
**#6**	**Limits:** trials; without limitation of language or year of publication.	2017: 9

SUPPLEMENTARY MATERIAL 7: Search results EMBASE

Number of localized studies: 56
Number of studies after applying limits: 6

	**Descriptors**	Number of studies reached
**#1**	adult **OR** adults **OR** human **OR** humans	19,433,834
**#2**	“Transcutaneous Electric Nerve Stimulation” **OR** “Electrical Stimulation, Transcutaneous” **OR** “Stimulation, Transcutaneuos Electrical” **OR** “Transcutaneous Electrical Stimulation” **OR** “Percutaneuos Electric Nerve Stimulation” **OR** “Transdermal Electrostimulation” **OR** “Electrostimulation, Transdermal” **OR** TENS **OR** “Transcutaneous Electrical Nerve Stimulation” **OR** “Transcutaneous Nerve Stimulation” **OR** “Nerve Stimulation, Transcutaneuos” **OR** “Stimulation, Transcutaneous Nerve” **OR** “Electric Stimulation, Transcutaneous” **OR** “Stimulation, Transcutaneous Electric” **OR** “Transcutaneous Electric Stimulation” **OR** “Percutaneuos Electrical Nerve Stimulation” **OR** “Analgesic Cutaneous Electrostimulation” **OR** “Cutaneous Electrostimulation, Analgesic” **OR** “Electrostimulation, Analgesic Cutaneous” **OR** Electroanalgesia	18,273
**#3**	“randomized controlled trial” OR “controlled clinical trial” OR “randomized controlled trials”/exp OR “random allocation”/exp OR “double blind method”/exp OR “single blind method”/exp OR “clinical trial” OR “clinical trials”/exp OR (clinical* AND trial*) OR single* OR double* OR treble* OR triple* OR placebos/exp OR placebo* OR random* OR “research design”/exp OR “comparative study”/exp OR “evaluation studies”/exp OR (follow-up stud*/exp) OR (prospective stud*/exp) OR control* OR prospectiv* OR volunteer* NOT animal NOT (human AND animal)	11,131,437
**#4**	Chemokines **OR** “Cytokines, Chemotactic” **OR** Intercrines **OR** “Chemotactic Cytokines” **OR** cytokines **OR** cytokine.	522,171
**#5**	**#1** AND **#2** AND **#3** AND **#4**	56
**#6**	Limits: #5 AND 'human'/de AND 'article'/it AND ([adult]/lim OR [aged]/lim OR [middle aged]/lim)	6

SUPPLEMENTARY MATERIAL 8

List of excluded articles of the review and the reasons (n=315)
(First level: reading title and abstracts)

	Reference	Reason for exclusion
1	Abelson et al., (1983)	Without dosage cytokines (primary outcome)
2	Adedoyin et al., (2005)	TENS associated with other physiotherapeutic resources
3	Aghamohammadi et al., (2011)	Without dosage cytokines (primary outcome)
4	Akyuz et al., (1993)	Without dosage cytokines (primary outcome)
5	Alfieri V et al., (1987)	Without dosage cytokines (primary outcome)
6	Allais G et al., (2003)	Without dosage cytokines (primary outcome)
7	Al-Smadi J et al., (2003)	Without dosage cytokines (primary outcome)
8	Altay F et al., (2010)	Without dosage cytokines (primary outcome) and exercises associated with TENS in the experimental group.
9	Alves Silverio et al., (2015)	Without dosage cytokines (primary outcome)
10	Amer-Cuenca et al., (2011)	Without dosage cytokines (primary outcome)
11	Anderson and Lipscomb (1989)	Without dosage cytokines (primary outcome)
12	Anderson et al., (2004)	Without dosage cytokines (primary outcome)
13	Angulo and Colwell, Jr. (1990)	Without dosage cytokines (primary outcome)
14	Annal et al., (1992)	Without dosage cytokines (primary outcome)
15	Arai et al., (2008)	Without dosage cytokines (primary outcome) and group control
16	Askin, A., et al. (2014)	Not have intervention group with TENS
17	Atamaz, F. C., et al. (2012)	Without dosage cytokines (primary outcome)
18	Baeumler, P. I., et al. (2015)	Without dosage cytokines (primary outcome)
19	Barbarisi, M., et al. (2010)	Without dosage cytokines (primary outcome)
20	Baskurt, Z., et al. (2006).	Without dosage cytokines (primary outcome)
21	Bayindir, O., et al. (1991)	Without dosage cytokines (primary outcome)
22	Benedetti, F., et al. (1997)	Without dosage cytokines (primary outcome)
23	Bennett, M. I., et al. (2010	Without dosage cytokines (primary outcome)
24	Bernateck, M., et al. (2008)	Without dosage cytokines (primary outcome) and proposed control group (acupuncture required when the intervention is electroacupuncture)
25	Bertalanffy, A., et al. (2005)	Without dosage cytokines (primary outcome)
26	Besson, J. M. (1997)	Not have intervention group with TENS and not clinical trial

	Reference	Reason for exclusion
27	Bicer et al., (2005)	Not have intervention group with TENS
28	Binder et al., (2011)	Without dosage cytokines (primary outcome)
29	Borjesson et al., (1997)	Without dosage cytokines (primary outcome)
30	Bouguen et al., (2014)	Without dosage cytokines (primary outcome)
31	Bower et al., (1998)	Without dosage cytokines (primary outcome)
32	Breit and Van der Wall (2004)	Without dosage cytokines (primary outcome)
33	Bundsen et al., (1982)	Without dosage cytokines (primary outcome)
34	Burch et al., (2008)	Without dosage cytokines (primary outcome) proposed control group (TENS placebo required when the intervention is TENS)
35	Burssens et al., (2005)	Without dosage cytokines (primary outcome) and evaluation results by histology
36	Carman and Roach (1988)	Children population
37	Casale et al., (2013).	Without dosage cytokines (primary outcome) proposed control group (TENS placebo required when the intervention is TENS)
38	Cekmen et al., (2007)	Without dosage cytokines (primary outcome)
39	Cetin et al., (2008)	Without dosage cytokines (primary outcome) and association of physiotherapeutic resources
40	Chaidemenos et al., (2007)	Not have intervention group with TENS
41	Chan et al., (2015).	Without dosage cytokines (primary outcome)
42	Chao et al., (2007).	Without dosage cytokines (primary outcome)
43	Chee and Walton (1986)	Without dosage cytokines (primary outcome)
44	Cheing et al., (2003)	Without dosage cytokines (primary outcome)
45	Cheing et al., (2002)	Without dosage cytokines (primary outcome)
46	Cheing and Hui-Chan (2004)	Without dosage cytokines (primary outcome)
47	Cheing and Luk (2005)	Without dosage cytokines (primary outcome)
48	Chen et al., (2005)	Children population
49	Chen et al., (1998)	Without dosage cytokines (primary outcome)
50	Chen et al., (2007)	Without dosage cytokines (primary outcome)
51	Chen et al., (2015).	Animal study
52	Chen et al., (2017)	Article notes
53	Cherian et al., (2016)	Without dosage cytokines (primary outcome)

	Reference	Reason for exclusion
54	Chesterton et al., (2013)	Without dosage cytokines (primary outcome)
55	Chitsaz et al., (2009)	Without dosage cytokines (primary outcome)
56	Chiu et al., (1999)	Without dosage cytokines (primary outcome)
57	Cho et al., (2011)	Without dosage cytokines (primary outcome)
58	Cho et al., (2013)	Without dosage cytokines (primary outcome)
59	Conn et al., (1986)	Without dosage cytokines (primary outcome)
60	Coletta, R., et al. (1988)	Without dosage cytokines (primary outcome)
61	Coura et al., (2011)	Without dosage cytokines (primary outcome)
62	Craig et al., (1996)	Without dosage cytokines (primary outcome)
63	Crew et al., (2007)	Not have intervention group with TENS
64	Crompton et al., (1992)	Without dosage cytokines (primary outcome)
65	Cui et al., (2011).	Without dosage cytokines (primary outcome)
66	Da Silva et al., (2008)	Without dosage cytokines (primary outcome) and comparison of TENS versus hydrotherapy
67	Dawood and Ramos (1990).	Without dosage cytokines (primary outcome)
68	de Giorgi et al., (2017)	Without dosage cytokines (primary outcome)
69	de Jong et al., (2013)	Without dosage cytokines (primary outcome)
70	de Orange et al., (2003)	Without dosage cytokines (primary outcome)
71	de Paiva Tosato et al., (2007)	Without dosage cytokines (primary outcome)
72	de Sousa et al., (2014)	Without dosage cytokines (primary outcome)
73	Denegar and Perrin (1992)	Without dosage cytokines (primary outcome) and association of physiotherapeutic resources
74	Derosa et al., (2013)	Not have intervention group with TENS
75	Desantana et al., (2008)	Without dosage cytokines (primary outcome)
76	Desantana et al., (2009)	Without dosage cytokines (primary outcome)
77	di Benedetto et al., (1993)	Without dosage cytokines (primary outcome)
78	Dissanayaka et al., (2016)	Without dosage cytokines (primary outcome) and association of physiotherapeutic resources
79	Dobsak et al., (2006)	Without dosage cytokines (primary outcome) and not have intervention group with TENS
80	Dunn et al., (1989)	Without dosage cytokines (primary outcome)

	Reference	Reason for exclusion
81	Dusunceli et al., (2009)	Without dosage cytokines (primary outcome) and association of physiotherapeutic resources
82	Ekblom and Hansson (1985)	Without dosage cytokines (primary outcome)
83	El-Dawlatly et al., (2008)	Without dosage cytokines (primary outcome)
84	Erdogan et al., (2005)	Without dosage cytokines (primary outcome)
85	Esteban González et al., (2015)	Without dosage cytokines (primary outcome)
86	Eyigor et al., (2010)	Without dosage cytokines (primary outcome)
87	Fagade and Obilade (2003)	Without dosage cytokines (primary outcome)
88	Farina et al., (2004)	Without dosage cytokines (primary outcome)
89	Fassoulaki et al., (1993)	Without dosage cytokines (primary outcome)
90	Finsen et al., (1988)	Without dosage cytokines (primary outcome)
91	Fiorelli et al., (2016)	Not have intervention group with TENS
92	Forst et al., (2004)	Without dosage cytokines (primary outcome)
93	Forster et al., (1994)	Without dosage cytokines (primary outcome)
94	Gademan et al., (2013)	Without dosage cytokines (primary outcome)
95	Gadsby et al., (1997)	Without dosage cytokines (primary outcome)
96	Galli et al., (2015)	Without dosage cytokines (primary outcome)
97	Gemignani et al., (1991)	Without dosage cytokines (primary outcome)
98	Ghoname et al., (1999)	Without dosage cytokines (primary outcome)
99	Gilbert et al., (1986)	Without dosage cytokines (primary outcome)
100	Gollob et al., (2008)	Not have intervention group with TENS
101	Gossrau et al., (2011).	Without dosage cytokines (primary outcome)
102	Graff-Radford et al., (1989)	Without dosage cytokines (primary outcome)
103	Gregorini et al., (2010)	Without dosage cytokines (primary outcome)
104	Gurgen et al., (2014)	Animal study
105	Halle et al., (1986)	Without dosage cytokines (primary outcome)
106	Hamza et al., (1999)	Without dosage cytokines (primary outcome)

	Reference	Reason for exclusion
107	Hansson and Ekblom (1983)	Without dosage cytokines (primary outcome)
108	Hargreaves and Lander (1989)	Without dosage cytokines (primary outcome)
109	Harrison et al., (1986)	Without dosage cytokines (primary outcome)
110	Hart et al., (2012)	Without dosage cytokines (primary outcome)
111	Hashim et al, (2015)	Not have intervention group with TENS
112	Herman et al., (1994)	Without dosage cytokines (primary outcome)
113	Herrera-Lasso et al., (1993)	Without dosage cytokines (primary outcome)
114	Hershman et al., (1989)	Without dosage cytokines (primary outcome)
115	Hettrick et al., (2004)	Without dosage cytokines (primary outcome)
116	Hidderley and Weinel (1997)	Without dosage cytokines (primary outcome)
117	Ho et al., (1989)	Without dosage cytokines (primary outcome)
118	Hruby et al., (2006)	Without dosage cytokines (primary outcome)
119	Hsieh and Lee (2002)	Without dosage cytokines (primary outcome)
120	Huang et al., (2008)	Not have intervention group with TENS
121	Itoh et al., (2008)	Without dosage cytokines (primary outcome)
122	Jensen et al., (1991)	Without dosage cytokines (primary outcome)
123	Johansson et al., (2001)	Without dosage cytokines (primary outcome)
124	Jones and Hutchinson (1991)	Without dosage cytokines (primary outcome)
125	Jung et al., (2016)	Without dosage cytokines (primary outcome)
126	Kara et al., (2010)	Without dosage cytokines (primary outcome)
127	Kaya et al., (2012	Not have intervention group with TENS
128	Keskin et al., (2012)	Without dosage cytokines (primary outcome)
129	Khan et al., (2005)	Without dosage cytokines (primary outcome)
130	Kim et al., (2014)	Without dosage cytokines (primary outcome)
131	Kim et al., (2013)	Without dosage cytokines (primary outcome)
132	Kim Y. et al., (2014)	Not have intervention group with TENS

	Reference	Reason for exclusion
133	Kirtsreesakul et al., (2016)	Not have intervention group with TENS
134	Knowles et al., (2015)	Without dosage cytokines (primary outcome)
135	Koh et al., (2013)	Without dosage cytokines (primary outcome) and association of physiotherapeutic resources
136	Korkmaz et al., (2010)	Without dosage cytokines (primary outcome)
137	Kruger et al., (1998)	Without dosage cytokines (primary outcome)
138	Labrecque et al., (1999)	Without dosage cytokines (primary outcome)
139	Laddha et al., (2016)	Without dosage cytokines (primary outcome)
140	Laitinen and Nuutinen (1991)	Without dosage cytokines (primary outcome)
141	Langley et al., (2010)	Without dosage cytokines (primary outcome)
142	Lau and Jones (2008)	Without dosage cytokines (primary outcome)
143	Lauretti et al., (2015)	Without dosage cytokines (primary outcome)
144	Law and Cheing (2004)	Without dosage cytokines (primary outcome)
145	Law et al., (2004)	Without dosage cytokines (primary outcome)
146	Leandri et al., (1990)	Without dosage cytokines (primary outcome)
147	Lee et al., (2015).	Without dosage cytokines (primary outcome)
148	Lee et al., (1990)	Without dosage cytokines (primary outcome)
149	Lehmann et al., (1983)	Without dosage cytokines (primary outcome)
150	Leo et al., (1986)	Without dosage cytokines (primary outcome)
151	Levin and Hui-Chan (1992)	Without dosage cytokines (primary outcome)
152	Lewers et al., (1989).	Without dosage cytokines (primary outcome)
153	Lewis et al., (2015)	Without dosage cytokines (primary outcome)
154	Lewis et al., (1990)	Without dosage cytokines (primary outcome)
155	Likar et al., (2001).	Without dosage cytokines (primary outcome)
156	Lima et al., (2011)	Without dosage cytokines (primary outcome)
157	Limoges and Rickabaugh (2004)	Without dosage cytokines (primary outcome)
158	Lin et al., (2010)	Children population

	Reference	Reason for exclusion
159	Liu et al., (2016)	Without dosage cytokines (primary outcome)
160	Liu et al., (1985)	Without dosage cytokines (primary outcome)
161	Lofgren and Norrbrink (2009)	Without dosage cytokines (primary outcome)
162	Long et al., (2002)	Not have intervention group with TENS
163	Longobardi et al., (1989)	Without dosage cytokines (primary outcome)
164	López-Pousa et al., (2015)	Not have intervention group with TENS
165	Lordelo et al., (2010)	Children population
166	Lorenzana (1999)	Without dosage cytokines (primary outcome)
167	Luijpen et al., (2004)	Without dosage cytokines (primary outcome)
168	Lundeberg (1984)	Without dosage cytokines (primary outcome)
169	Lundeberg (1984).	Without dosage cytokines (primary outcome)
170	Lundeberg et al. (1985)	Without dosage cytokines (primary outcome)
171	Maayah and al-Jarrah (2010).	Without dosage cytokines (primary outcome)
172	Mankovsky-Arnold et al., (2013)	Without dosage cytokines (primary outcome)
173	Mannheimer and Carlsson (1979)	Without dosage cytokines (primary outcome)
174	Mannheimer et al., (1985)	Without dosage cytokines (primary outcome)
175	Mannheimer et al., (1985)	Without dosage cytokines (primary outcome)
176	Mannheimer et al., (1986)	Without dosage cytokines (primary outcome)
177	Mannheimer et al., (1978)	Without dosage cytokines (primary outcome)
178	Mannheimer and Whalen (1985)	Without dosage cytokines (primary outcome)
179	Manning et al., (2014)	Not have intervention group with TENS
180	Martins et al., (2012)	Without dosage cytokines (primary outcome)
181	Mascarin et al., (2012)	Without dosage cytokines (primary outcome)
182	McCallum et al., (1988)	Without dosage cytokines (primary outcome)
183	Meamarbashi and Rajabi (2015)	Not have intervention group with TENS
184	Melo de Paula et al., (2006)	Without dosage cytokines (primary outcome)

	Reference	Reason for exclusion
185	Miller et al., (2007).	Without dosage cytokines (primary outcome)
186	Mills et al., (2014)	Not have intervention group with TENS
187	Milsom et al., (1994)	Without dosage cytokines (primary outcome)
188	Mira et al., (2015)	Without dosage cytokines (primary outcome)
189	Moeller Joensson et al., (2015)	Children population
190	Moniruzzaman et al., (2010)	Without dosage cytokines (primary outcome)
191	Mora et al., (2006)	Without dosage cytokines (primary outcome)
192	Mucuk et al., (2013)	Without dosage cytokines (primary outcome)
193	Murina et al., (2008).	Without dosage cytokines (primary outcome)
194	Murray et al., (2004)	Without dosage cytokines (primary outcome)
195	Navarathnam et al., (1984)	Without dosage cytokines (primary outcome)
196	Neighbors et al., (1987)	Without dosage cytokines (primary outcome)
197	Ng et al., (2003)	Without dosage cytokines (primary outcome)
198	Ng and Hui-Chan (2007)	Without dosage cytokines (primary outcome)
199	Ng and Hui-Chan (2009)	Without dosage cytokines (primary outcome)
200	Ngai et al., (2009)	Without dosage cytokines (primary outcome)
201	Ni, X., et al. (2012)	Children population
202	Nigam et al., (2011)	Not have intervention group with TENS
203	Nilsson et al., (2004)	Not have intervention group with TENS
204	Oncel et al., (2002)	Without dosage cytokines (primary outcome)
205	Ordog (1987)	Without dosage cytokines (primary outcome)
206	O'Reilly et al., (2008)	Without dosage cytokines (primary outcome)
207	Ou et al., (2012)	Not have intervention group with TENS
208	Ozturk et al., (2016)	Without dosage cytokines (primary outcome)
209	Paker et al., (2006)	Without dosage cytokines (primary outcome)
210	Paoloni and Murrell (2007)	Not have intervention group with TENS

	Reference	Reason for exclusion
211	Park et al., (2014).	Without dosage cytokines (primary outcome)
212	Paternostro-Sluga et al., (1999).	Without dosage cytokines (primary outcome)
213	Pearl et al., (1999)	Without dosage cytokines (primary outcome)
214	Perissinotto et al., (2015)	Without dosage cytokines (primary outcome)
215	Picelli et al., (2014).	Without dosage cytokines (primary outcome)
216	Pietrosimone et al., (2009)	Without dosage cytokines (primary outcome)
217	Pietrosimone et al., (2010)	Without dosage cytokines (primary outcome)
218	Pietrosimone et al., (2011).	Without dosage cytokines (primary outcome)
219	Pimenta Amaral et al., (2012)	Without dosage cytokines (primary outcome)
220	Pitangui et al., (2012)	Without dosage cytokines (primary outcome)
221	Plaster et al., (2014)	Without dosage cytokines (primary outcome)
222	Prabhakar and Ramteke (2011)	Without dosage cytokines (primary outcome)
223	Quinton et al., (1987)	Without dosage cytokines (primary outcome)
224	Rajpurohit et al., (2010)	Without dosage cytokines (primary outcome)
225	Rakel et al., (2014)	Without dosage cytokines (primary outcome)
226	Rakel and Frantz (2003)	Without dosage cytokines (primary outcome)
227	Ratajczak et al., (2011)	Without dosage cytokines (primary outcome)
228	Reichstein et al., (2005)	Without dosage cytokines (primary outcome)
229	Reuss et al., (1988)	Without dosage cytokines (primary outcome)
230	Robinson et al., (2001)	Without dosage cytokines (primary outcome)
231	Roche et al., (1985)	Without dosage cytokines (primary outcome)
232	Rodriguez-Fernandez et al., (2011)	Without dosage cytokines (primary outcome)
233	Rogge (2002)	Not have intervention group with TENS
234	Rooney et al., (1983)	Without dosage cytokines (primary outcome)
235	Rorsman and Johansson (2006)	Without dosage cytokines (primary outcome)
236	Rosenberg et al., (1978)	Without dosage cytokines (primary outcome)

	Reference	Reason for exclusion
237	Rusconi et al., (2002)	Without dosage cytokines (primary outcome)
238	Saal (1996)	Not have intervention group with TENS
239	Sahin et al., (2011)	Without dosage cytokines (primary outcome)
240	Santana et al., (2016)	Without dosage cytokines (primary outcome)
241	Scherder and Bouma (1999)	Without dosage cytokines (primary outcome)
242	Scherder et al., (1998).	Without dosage cytokines (primary outcome)
243	Scherder et al., (1999)	Without dosage cytokines (primary outcome)
244	Schmidt et al., (2012)	Not have intervention group with TENS
245	Schreiner et al., (2010)	Without dosage cytokines (primary outcome)
246	Schroder et al., (2008)	Without dosage cytokines (primary outcome)
247	Schuhfried et al., (2005)	Without dosage cytokines (primary outcome)
248	Schuster and Infante (1980)	Without dosage cytokines (primary outcome)
249	Sela and Mozes (2004)	Not have intervention group with TENS
250	Sencan et al., (2004)	Without dosage cytokines (primary outcome)
251	Shamley et al., (2009)	Not have intervention group with TENS
252	Shehab and Adham (2000)	Without dosage cytokines (primary outcome)
253	Silva et al., (2012)	Without dosage cytokines (primary outcome)
254	Sim (1991)	Without dosage cytokines (primary outcome)
255	Smania et al., (2005)	Without dosage cytokines (primary outcome)
256	Smedley et al., (1988)	Without dosage cytokines (primary outcome)
257	Smith et al., (1986	Without dosage cytokines (primary outcome)
258	Sodipo et al., (1980)	Without dosage cytokines (primary outcome)
259	Solak et al., (2009)	Without dosage cytokines (primary outcome)
260	Sonde et al., (2000)	Without dosage cytokines (primary outcome)
261	Soomro et al., (2001)	Without dosage cytokines (primary outcome)
262	Souto et al., (2014)	Without dosage cytokines (primary outcome)

	Reference	Reason for exclusion
263	Stratton and Smith (1980)	Without dosage cytokines (primary outcome)
264	Stubbing, and Jellicoe (1988)	Without dosage cytokines (primary outcome)
265	Surbala et al., (2014)	Without dosage cytokines (primary outcome)
266	Tam et al., (2002)	Not have intervention group with TENS
267	Tarcin et al., (2004)	Not have intervention group with TENS
268	Taylor et al., (1981)	Without dosage cytokines (primary outcome)
269	Tekeoglu et al., (1998)	Without dosage cytokines (primary outcome)
270	Thakur and Rekha (2004)	Without dosage cytokines (primary outcome)
271	Thomas et al., (1988)	Without dosage cytokines (primary outcome)
272	Thomas et al., (1995)	Without dosage cytokines (primary outcome)
273	Thomsen et al., (2002)	Not have intervention group with TENS
274	Tilak et al., (2016)	Without dosage cytokines (primary outcome)
275	Tiplica and Salavastru (2009)	Not have intervention group with TENS
276	Tokuda et al., (2014)	Without dosage cytokines (primary outcome)
277	Tonella et al., (2006)	Without dosage cytokines (primary outcome)
278	Toyota et al., (1999)	Without dosage cytokines (primary outcome)
279	Tsang et al., (2011)	Without dosage cytokines (primary outcome)
280	Tsen et al., (2000)	Without dosage cytokines (primary outcome)
281	Tsen et al., (2001)	Without dosage cytokines (primary outcome)
282	Tsukayama et al., (2002)	Without dosage cytokines (primary outcome)
283	Tugay et al., (2007)	Without dosage cytokines (primary outcome)
284	Tulgar et al., (1991)	Without dosage cytokines (primary outcome)
285	Tuzun et al., (2004)	Without dosage cytokines (primary outcome)
286	Tyson et al., (2013)	Without dosage cytokines (primary outcome)
287	Uebelhack et al., (2014)	Not have intervention group with TENS
288	Unterrainer et al., (2012)	Without dosage cytokines (primary outcome)

	Reference	Reason for exclusion
289	Valenza et al. (2016)	Without dosage cytokines (primary outcome)
290	Van der Peijl et al., (1998)	Without dosage cytokines (primary outcome)
291	Van der Ploeg et al., (1996)	Without dosage cytokines (primary outcome)
292	Van Someren et al., (1998)	Without dosage cytokines (primary outcome)
293	Vance et al., (2012)	Without dosage cytokines (primary outcome)
294	Walker et al., (1991)	Without dosage cytokines (primary outcome)
295	Wang et al., (2007)	Without dosage cytokines (primary outcome)
296	Wang et al., (2007)	Without dosage cytokines (primary outcome)
297	Warfield et al., (1985)	Without dosage cytokines (primary outcome)
298	Warke et al., (2004)	Without dosage cytokines (primary outcome)
299	Warner et al., (2013)	Without dosage cytokines (primary outcome)
300	Wei et al., (2016)	Without dosage cytokines (primary outcome)
301	Weng et al., (2005)	Without dosage cytokines (primary outcome)
302	Weng et al., (2005)	Without dosage cytokines (primary outcome)
303	Widerstrom-Noga et al., (1998)	Without dosage cytokines (primary outcome)
304	Wong et al., (2010)	Not have intervention group with TENS
305	Wu et al., (2016)	Not have intervention group with TENS
306	Wu et al., (2015)	Without dosage cytokines (primary outcome)
307	Xu et al., (2007)	Children population
308	Yameen et al., (2011)	Without dosage cytokines (primary outcome)
309	Yazmalar et al., (2016)	Without dosage cytokines (primary outcome)
310	Yeh et al., (2015)	Without dosage cytokines (primary outcome)
311	Yilmazer et al., (2012)	Without dosage cytokines (primary outcome)
312	Zhang et al., (2014)	Without dosage cytokines (primary outcome)
313	Zhao et al., (2012)	Not have intervention group with TENS
314	Zhao et al., (2015)	Not have intervention group with TENS
315	Zhu et al., (2013)	Not have intervention group with TENS

SUPPLEMENTARY MATERIAL 9

List of excluded articles of the review and the reasons (n=26)
(Second level: reading the full text)

	Reference	Reason for exclusion
1	Amaral et al., (2012)	Dosage proinflammatory cytokines in salivary flow
2	Chen et al., (2012)	Not evaluate the TENS.
3	Chen et al., (2015)	Not present the proposed correspondence between intervention group and control group; for electroacupuncture, acupuncture is required as a control.
4	de Angelis et al., (2003)	Hemodynamic parameters were evaluated but among them we did´t find the dosage of proinflammatory cytokines.
5	Feng and Li (2013)	Not present the proposed correspondence between intervention group and control group; for electroacupuncture, acupuncture is required as a control.
6	Franco et al., (2014)	Use laboratory tests as outcome, but not found the dosage of proinflammatory cytokines (primary outcome)
7	Frasko et al., (2008)	Electrogastrography is a diagnostic method; Not have a intervention group TENS
8	Geirsson et al., (1993)	Comparison of TENS with acupuncture (Not present the proposed correspondence between intervention group and control group); Without dosage of proinflammatory cytokines.
9	Grech et al., (2016)	Not present the proposed correspondence between intervention group and control group; for electroacupuncture, acupuncture is required as a control.
10	Han et al., (2016)	Not present the proposed correspondence between intervention group and control group; for electroacupuncture, acupuncture is required as a control. They will use needles that do not penetrate the skin in acupoints.
11	Jong et al., (2006)	Not present the proposed correspondence between intervention group and control group; for electroacupuncture, acupuncture is required as a control.
12	Lerman et al., (2016)	They used the electrical current but not the current studied.<br>Not have a intervention group TENS
13	McNearney et al., (2013)	The study was an open-label design; all patients received the same therapy and acted as their own controls; does not satisfy the criterion of at least one group control and an intervention. (not RCT)
14	Qiu et al., (2007)	Not present the proposed correspondence between intervention group and control group; for electroacupuncture, acupuncture is required as a control.
15	Shen et al., (2011)	Not present the proposed correspondence between intervention group and control group; for electroacupuncture, acupuncture is required as a control.
16	Song et al., (2009)	Not present the proposed correspondence between intervention group and control group; for electroacupuncture, acupuncture is required as a control. In this case the sham acupunture was made in different acupoints of the eletroacupunture group.

17	Stavrakis et al., (2014)	Not have a intervention group TENS; They used Transcutaneous low-level tragus electrical stimulation (flat metal clip onto the tragus produced the cureent in the ear)
18	Teodorczyk-Injeyan et al., (2015)	Not have a intervention group TENS; eles testam um aparelho (InterX5000) with objetive the shown that interactive neurostimulation therapy may be also efficacious for pain compared with transcutaneous electrical nerve stimulation (TENS).
19	Wang et al., (2007)	Not present the proposed correspondence between intervention group and control group; for electroacupuncture (EA), acupuncture is required as a control. In addition warming therapy combined with EA.
20	Wang X (2007)	Despite having the dosage of proinflammatory cytokines, not present the proposed correspondence between intervention group and control group; for electroacupuncture (EA), acupuncture is required as a control.
21	Wu et al., (2010)	Not present the proposed correspondence between intervention group and control group; for electroacupuncture, acupuncture is required as a control.
22	Xu, F. Y., et al. (2009)	Not randomised clinical trial (RCT)
23	Yan et al., (2014)	Not present the proposed correspondence between intervention group and control group; for electroacupuncture, acupuncture is required as a control.
24	Yang et al., (2003)	Not evaluate the TENS.
25	Zhang et al., (2015)	Electroacupuncture combined with ultraviolet therapy
26	Zhu et al., (2015)	Not present the proposed correspondence between intervention group and control group; for electroacupuncture, acupuncture is required as a control.

SUPPLEMENTARY MATERIAL 10

List of excluded research in clinical trial repositories

**Registros Brasileiros de Ensaios Clínicos** (Search term: Estimulação elétrica nervosa transcutânea)		
	**Identifier**	**Reason for exclusion**
1	*RBR-4wfh7y (2015)*	Without dosage cytokines (primary outcome)
2	RBR-3rndh6 (2013)	Without dosage cytokines (primary outcome)
3	*RBR-8xtkjp (2012)*	Without dosage cytokines (primary outcome)
4	*RBR-459y54 (2013)*	Without dosage cytokines (primary outcome)
5	*RBR-8jg3bk (2014)*	Without dosage cytokines (primary outcome)
6	*RBR-8ftzft (2012)*	Without dosage cytokines (primary outcome)
**Clinical Trials** (Search term: transcutaneous electrical nerve stimulation and cytokines)		
	**ClinicalTrials.gov Identifier**	**Reason for exclusion**
1	NCT02548754 (2015)	Still in recruitment; Not response from contact with the authors.
2	NCT02898181 (2016)	Still in recruitment; Not response from contact with the authors.
3	NCT02910973 (2016)	Still in recruitment; Not response from contact with the authors.
4	NCT02813629 (2016)	Not have intervention group with TENS

SUPPLEMENTARY MATERIAL 11: Description of other characteristics of the studies.

Author (year)	Objectives	Descrição da amostra			Intervention				Outcomes	
		Clinical diagnosis	Speciality	Eligibility	Application	Treatment duration/ session	Details Experimental group	Details Control group	Description principal results	Pain (secondary)
Fiorelli et al. (2012)[6]	We aimed to assess the efficacy of TENS on post-thoracotomy pain in relation of four criterion measurements as: (i) cytokines; (ii) pain; (iii) respiratory function and (iv) intake of narcotic medication.	Lung Cancer	Oncology	Series of consecutive patients undergoing standard posterolateral thoracotomy for pulmonary resection.Exclusion criteria were: (i) previous history of chronic pain; (ii) preoperative use of narcotic and/or of TENS;(iii) previous thoracic procedure; (iv) presence of pacemaker; (v)neurologic disease such as movement limitation or cerebral confusion and (vi) other types of incision different from posterolateral thoracotomy.	Placed on the skin on either dorsal side of the incision 2 cm away the suture line.	TENS immediately started and were performed at intervals of 4h each, all with a duration of 30 min in the first 48 POHs. Then, TENS was applied twice daily up to 5 postoperative days	The TENS group adjusted the stimulus intensity until a strong but comfortable tingling sensation was felt	Whereas the placebo TENS group was told that the electrical stimulation was silent, producing no sensation. However, in the placebo group the TENS unit also displayed an active indicator light, suggesting to the patient that the unit was active.	Serum IL-6 (P = 0.001), IL-10 (P = 0.001) and TNF-α (P = 0.001) levels in TENS group were significantly lower than in the control group;	VAS score in TENS group was significantly lower than in the control group (P < 0.001)
Ngai et al. (2010)[41]	This study investigated the effect of 4-week Acu-TENS on physical and psychosocial function in these patients.	Chronic obstructive pulmonary disease (COPD)	Pneumology	Patients with diagnosis of COPD based on the GOLD guideline and attending regular follow-up at the respiratory clinic of a local district hospital, were invited to participate in the study. COPD was defined as—FEV1/FVC ratio < 70%, with an increase in FEV1 < 200 ml and <15% of the baseline value, after bronchodilator administration. Patients who were unable to perform spirometry testing, confused and/or incommunicable, allergic to aqueous gel, or suffering from upper respiratory tract infection or COPD exacerbation in the past 6 weeks and/or having musculoskeletal disorders that might affect their performance in 6-min walk test (6MWT), were excluded	Dingchuan (EX-B1)	A "course" of acupuncture or Acu-TENS has been reported to Accordingly, this study adopted a 4-week program of 5 sessions per week consist of 20 treatment sessions with duration 45 minutes.	Acu-TENS—TENS applied bilaterally over the acupuncture points(EX-B1, Dingchuan), 0.5 cun lateral to the lower border of the 7th cervical vertebra, whereа 'cun' is the distance between the medial ends of the creases of the interphalangeal joints of the subjects' middle finger.	Placebo-TENS—identical to Acu-TENS but with no electrical output from the machine, despite an activated output display screen.	"IL-8 was hardly detectable in all subject groups, while CRP was detected in 20 and TNF- in 9 subjects. Changes in immunological markers were not significant in any of the three intervention groups."	not avaliable
OUYANG et al. (2010)[43]	To explore the mechanism of electro acupuncture on rheumatoid arthritis (RA).	Rheumatoid Arthritis	Rheumatology	**Inclusion**: 1. meet the diagnostic criteria for active RA; 2. aged 18 to 70 years old, both men and women; 3.informed consent, voluntary test, and signed informed consent. **Exclusion**: 1. pregnant women, pregnant women and lactating women; 2. severe heart, liver, renal insufficiency or systemic failure; 3.complicated by other rheumatoid diseases such as systemic lupus erythematosus, Sjogren's syndrome, severe knee osteoarthritis and other patients; 4.late patients, severe joint deformity, joint function grade Ⅳ; 5. not suitable for acupuncture treatment of other diseases.	acupoint: Baihui (DU20), Fengchi (GB20), Quchi (LI11), Waiguan (SJ6), Guanyuan (RN4), Zusanli (ST36), Yanglingquan (GB34), Xuanzhong (GB39), Sanyinjiao (SP6), Shenshu (BL23), Pishu (BL20).	The above method every other day treatment 1 times (30 min), 10 times for a course of treatment, after treatment of 3 courses observed efficacy.	In group of points, connect to the electroacupuncture instrument (EA apparatus type SDZ-Ⅱ), with continuous wave, adjust the current, the patient can tolerate and have a sense of comfort, retaining needle for 30 min.	Acupoints and acupuncture group are the same with the acupuncture group, but do not receive electro-acupuncture instrument.	IL-1 and IL-6 peripheral blood was better than that in electroacupuncture group compared with acupuncture group (P <0.05).	not avaliable
OUYANG et al. (2011)[42]	To observe the effect of electro-acupuncture (EA) on tumor necrosis factor-α (TNF-α) and vascular endothelial growth factor (VEGF) in peripheral blood and joint synovia in patients with rheumatoid arthritis (RA) to verify the clinical efficacy of EA.	Rheumatoid Arthritis	Rheumatology	The patients included in the trial were those (1) matched the diagnostic standard of RA at active stage; (2) 18–70 years old, no limitation of sex; and (3) who understand the trial and take part in it voluntary and signed the written informed consent.	Acupoints: Baihui (DU20), Fengchi (GB20), Quchi (LI11), Waiguan (SJ6), Guanyuan (RN4), Zusanli (ST36), Yanglingquan (GB34), Xuanzhong (GB39), Sanyinjiao (SP6), Shenshu (BL23), Pishu (BL20).	The treatment was carried out once every other day (30 min), for 10 times as one course; the effectiveness of treatment was evaluated after three courses of treatment.	EA apparatus type SDZ-, applied continuous wave, adjusted the power to make the stimulation adaptable or even give comfortable sensation to patient	SN group, they received simple needling only, same as that described in EA, but no EA stimulationwas applied.	P<0.05, compared with pre-treatment in the samegroup; P<0.05, compared with the SN group post-treatment	not avaliable
WANG et al. (2008)[44]	To observe the effect of transcutaneous acupoint electrical stimulation (TAES) on plasma ET、CGRP and serum IL-6, S100β during craniotomy	patients underwent elective surgery for cerebrospinal operation (meningioma, Glioma, acoustic neuroma, pituitary tumor)	Neurology	Exclusion criteria: severe intracranial pressure increased (abnormal abnormalities with CT showed a neutral shift of more than 5 mm); alcohol or drug dependence; Glass score (GCS) <15; heart, liver, kidney dysfunction in patients	Quchi, Zusanli and Sanyinjiao the two groups.	All patients were treated with anesthesia and acupoint stimulation (T0), intracranial operation for 1 h (T1), surgery (T2), 24 h after operation (T3), 48 h (T4)	After 30 minutes of anesthesia induction, select the two sides of the valley and Quchi, Zusanli and Sanyinjiao the two groups. LH 402 Han's acupoint irritant instrument	The control group was treated with conventional general anesthesia and without TAES.	IL-6 content: The treatment group was significantly lower in T1 (P <0.01); The control group increased at T1 (P <0.05). The level of IL-6 in T4 was significantly lower than that in control group (P <0.01), and higher than T3 in control group (P <0.05).	not avaliable
